# Descellement aseptique d'une prothèse totale de la hanche par fracture de la tige: un cas rare dans la littérature

**DOI:** 10.11604/pamj.2016.23.161.8873

**Published:** 2016-04-06

**Authors:** Adil El Alaoui, Ilyas Rabhi

**Affiliations:** 1 Service de Chirurgie Orthopédique du Centre Hospitalier de Chambéry, France

**Keywords:** Fracture, tige, prothèse, hanche, Fracture, stem, prosthesis, hip

## Image en médecine

Il s'agit d'un patient âgé de 82 ans, opéré il y a 15 ans pour coxarthrose de la hanche gauche pour laquelle il a bénéficié d'une prothèse totale de hanche avec une bonne évolution clinique et radiologique puis opéré il y a 10 ans pour le côté droit avec mise en place d'une prothèse totale de hanche. L’évolution était marquée au niveau du côté droit par la sensation des douleurs au niveau de la hanche avec une impotence fonctionnelle totale du membre inférieure gauche. L'examen clinique a permis de mettre en évidence un raccourcissement du membre inférieur droit estimé à 4 cm, avec une attitude en adduction et rotation externe. La radiographie du bassin a objectivé un descellement de la prothèse de hanche par fracture de la tige fémorale au niveau du col (A). Le patient a été réopéré pour ablation de la prothèse fracturée (B) et son changement par un autre type de prothèse cimentée. La radiographie de contrôle a montré une bonne position de la prothèse avec correction de la longueur du membre inférieure droit (C). Les suites post-opératoires étaient simples, avec une reprise de la marche et disparition des douleurs après 10 séances de rééducation.

**Figure 1:**
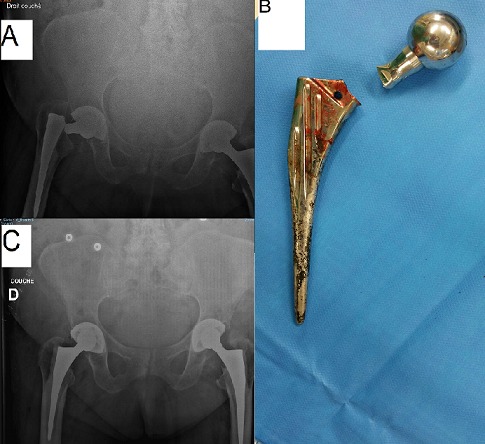
A): radiographie du bassin face montrant une fracture de la tige prothétique droite au niveau du col fémoral; B): image peropératoire de la tige fémorale fracturée; C): radiographie de contrôle du bassin montrant une bonne position de la nouvelle prothèse avec correction de la longueur du membre

